# 

**Published:** 1974-04

**Authors:** 


The
Bristol Medico-Chirurgical Journal
(Incorporating the Medical Journal of the South-West)
Established 1883

OCT 24 1975

OCT 31 1975

Vol. 89 (ii) APRIL 1974 No. 330

### BRISTOL MEDICO-CHIRURGICAL JOURNAL

Editor: N. J. Brown, M.B., F.R.C.P., F.R.C.Path.

Published Quarterly

Annual Subscription £2 Post Free

### BRISTOL MEDICO-CHIRURGICAL SOCIETY

**President 1973-74: Dr. R. F. Barbour**

**President-elect: Dr. J. Apley**

**Honorary Secretary: Dr. I. S. Bailey, 7 Percival Road, Bristol 8.**

**Honorary Treasurer: Dr. Frank Ross, Bristol Royal Infirmary, Bristol 2.**

The Society was founded in 1874. In 1883 publication of the Journal began and has continued quarterly ever since then. During the years 1949 to 1962 it appeared under the title of "The Medical Journal of the South West".

### THE BRISTOL MEDICO-CHIRURGICAL JOURNAL

**Editorial Committee**

| | |
|---|---|
| Editor | Dr. N. J. Brown, Southmead Hospital, Bristol. |
| Assistant Editor | Dr. D. S. Reeves. |
| Members | Dr. Rhys Davies.<br>Dr. M. B. Lennard.<br>Professor R. C. Wofinden.<br>Dr. D. W. Wright.<br>The Hon. Treasurer and Hon. Secretary of the Society. |
| Business Manager | Dr. P. J. F. Baskett, Frenchay Hospital, Bristol. |
| Secretary | Mr. B. P. Jones, The Library, Medical School, University Walk, Bristol BS8 1TD. |

### NOTICE TO CONTRIBUTORS

The Journal is especially concerned to provide a place for the recording of medical thought, interests, and practice in and around Bristol. It therefore welcomes articles that, as well as being of immediate interest to members, will document the local and contemporary medical scene for our successors.

Original articles are invited provided that they have not been published elsewhere. References in the text should be by author's name, with year of publication in parenthesis; they should be listed at the end in alphabetical order, the name of each journal or book being given in full—not abbreviated—and the title of the article added. Illustrations should be supplied ready for reproduction. Line drawings should be in Indian ink on firm white paper or board. The author will be informed if it is found necessary to ask him to pay for the making of blocks.

The following contributions will be especially welcome; notes of forthcoming events, meetings held, degrees conferred, staff appointments, honours and public appointments and other local news.

### The Medical Library

In 1890 the Bristol Medico-Chirurgical Society founded a medical library which in 1892 was combined with that of the Bristol Medical School. This became the University of Bristol Medical Library in 1925 and an agreement in that year confirmed the privilege of Members of the Society to use the University Medical Library.

### A Selection of Recent Additions to the Medical Library

ASSALI, N. S.: Pathophysiology of gestation. 3 vols. (Acad. Press)
BAHN, A. K.: Basic medical statistics. (Grune & Stratton)
BAILEY, G. L. ed.: Hemodialysis: principles and practice. (Acad. Press)
BARRATT-BOYES, B. G. et al.: Heart disease in infancy: diagnosis and surgical treatment. (Churchill Livingstone)
BERGMANN, K.: The aged: their understanding and care. (Wolfe)
BOYARSKY, S. & P. C. LABAY: Ureteral dynamics: pathophysiology, drugs and surgical implications. (Churchill Livingstone)
BROD, J.: The kidney. (Butterworths)
BRODER, L. E., & S. K. Carter: Meningeal leukaemia. (Plenum Press)
BURTON, J. L.: Aids to postgraduate medicine. (Churchill Livingstone)
BYRNE, P. S., & B. E. LONG: Learning to care: person to person. (Churchill Livingstone)
CIBA FOUNDATION: Atherogenesis: initiating factors. (Elsevier)
CIBA FOUNDATION: Intrauterine infections. (Elsevier)
CLARKE, E. & K. DEWHURST: An illustrated history of brain function. (Sandford Publications)
CLOUET, D. H. ed.: Narcotic drugs biochemical pharmacology. (Plenum Press)
CONN, H. F. et al eds.: Family practice. (Saunders)
COREY, L. et al. eds.: Medicine in a changing society. (Mosby)
DASH, C. H. & H. E. H. JONES: Mechanisms in drug allergy. (Churchill Livingstone)
DEICHMANN, W. B. & H. W. GERARDE: Toxicology of drugs and chemicals. (Acad. Press)
D'ERAMO, N. & M. LEVI: Neurological symptoms in blood diseases. (Harvey Miller & Medcalf)
EASSON, E. C.: Cancer of the uterine cervix. (Saunders)
EHRLICH, G. E. ed.: Total management of the arthritic patient. (Lippincott)
FOLDI, M.: Diseases of lymphatics and lymph circulation. (Thomas)
FRANKLIN, D. A. & G. B. NEWMAN: A guide to medical mathematics. (Blackwell)
FROMMER, E. A.: Diagnosis and treatment in clinical child psychiatry. (Heinemann)
FUSEK, I. & Z. KUNC eds.: Present limits of neurosurgery. (Excerpta Medica)
GARB, S.: Clinical guide to undesirable drug interactions and interferences. (Harvey Miller & Medcalf)
GOODGOLD, J. & A. EBERSTEIN: Electrodiagnosis of neuromuscular diseases. (Williams & Wilkins)
GORDON, R. A. et al eds.: Malignant hyperthermia. (Thomas)
GRAHAM, J. H. et al eds.: Dermal pathology. (Harper & Row)
HALL, T. A. et al: X-ray microscopy in clinical and experimental medicine. (Thomas)
HALL, T. C. ed.: Prediction of response in cancer therapy. (National Cancer Institute)
HALMOS, P.: The faith of the counsellors. (Constable)
HAMBURGER, J. I.: Hyperthyroidism: concept and controversy. (Thomas)
HARTLAGE, L. C. & D. G. LUCAS: Mental development: evaluation of the pediatric patient. (Thomas)
HEIMLICH, H. ed.: Surgery of the liver and intrahepatic bile ducts. (Hilger)
HERSH, E. M. et al: Immunotherapy of cancer in man: scientific basis and current status. (Thomas)
HOWARD, A. N. & I. M. BAIRD: Nutritional deficiencies in modern society. (Newman Books)
HOWELLS, T. H. & A. W. GROGNO eds.: Anaesthesia in organ transplantation. (Karger)
HUBBARD, C. W.: Family planning education: parenthood and social disease control. (Mosby)
HUGHES, E. S. R. & A. M. CUTHBERTSON: Anorectal surgery. (Chapman & Hall)
JACOB, A.: Clinical cardiac roentgen diagnosis. (Hilger)
JACOBSEN, E.: Depression: comparative studies of normal, neurotic and psychotic conditions. (International University Press)
JARRETT, A. ed.: The physiology and pathophysiology of the skin. 2 vols. (Acad. Press)
JONES, K. L. et al: Total fitness. (Canfield Press)
KAGAN, B. M. & E. R. STIEHM: Immunologic incompetence. (Year Book Med. Publ.)
KANNEL, W. B.: The natural history of myocardial infarction: the Framingham study. (Leiden Univ. Press)
KLINGBERG, M. A. et al eds.: Drugs and foetal development. (Plenum Press)
KOPEC, A. C.: Distribution of the blood groups in the United Kingdom. (Oxford Univ. Press)
KOSTERLITZ, H. W. et al eds.: Agonist and antagonist actions of narcotic analgesic drugs. (Macmillan)

### Bristol Medico-Chirurgical Society

The fourth meeting of the session was held at the Medical School on 9th January, 1974. Dr. A. F. Rogers showed a wall demonstration on the measurement of prostaglandins. The speaker was Sir Douglas Black, Chief Scientist to the Department of Health and formerly Professor of Medicine in the University of Manchester. His subject was 'The Professor of Medicine—Tod und Erklarung'.

At the fifth meeting on 13th February, Dr. C. R. Tribe showed pathological specimens and Dr. F. G. M. Ross demonstrated skiagrams. Professor C. Bruce Perry addressed the Society on 'The History of Medicine in Bristol and of the Bristol Medico-Chirurgical Society'. This paper will appear in the next issue of the Journal which will be a special number commemorating the Centenary of the Society.

At both meetings Mr. B. P. Jones arranged his usual interesting display of medical books.

### News and Notes

**University of Bristol**

M.D.—Jean James
F. J. Roberts

Ch.M.—B. H. Cummins
M. G. Johnson

Ph.D. (Faculty of Medicine)—R. F. Searle
R. W. Lacey

M.Sc. in Medical Sciences—R. S. Yilmaz

**NEW YEAR HONOURS**

**C.B.E.** G. H. Tovey, Director Regional Blood Transfusion Service and National Tissue Typing Reference Centre, Bristol.

**O.B.E.** G. Stewart Smith, formerly Consultant Pathologist, Exeter.

April 1st 1974

On 1st April 1974 the Re-Organisation of the National Health Service comes into effect. A detailed account of the new arrangements which have been widely publicised would be out of place in this Journal. A cynical reader, heavily involved in preparations for this event, has, however, felt moved to send in the following quotation:—

'We trained hard, but it seemed every time we were beginning to form into teams we would be reorganised. I was to learn later in life that we tend to meet every new situation by reorganising and a wonderful method it can be for creating the illusion of progress while producing confusion, inefficiency and demoralisation.'

Gaius Petronicus,
1st Century A.D.

### NOTICE

The Ninth International Congress of Chemotherapy will be held in London from July 13th to 18th, 1975 under the patronage of Her Majesty the Queen and the Presidency of Professor W. Brumfitt. Following the inaugural ceremony at the Albert Hall, the scientific sessions will be held at the Imperial College of Science and Technology. Chemotherapy embraces both antimicrobial and anticancer agents, so the special symposia will explore current research and new approaches in the management of infections and cancer —chemotherapy of tuberculosis, life-threatening infections, tropical diseases, antiviral and antifungal chemotherapy, venereal diseases, leukaemia, potentiators of radiotherapy, new anticancer regimes and immunological aspects of anticancer drugs. In addition to international symposia and invited special speakers, there are also sessions for free communications for which participants are invited to make 10 minute presentations followed by 5 minute discussion. The scientific sessions are supported by an outstanding social programme which includes sightseeing tours of London, Greenwich by river, a night out with London's Cockney Pearly Kings and Queens, and of course, trips to Hampton Court, Windsor Castle, Oxford, Blenheim, Chartwell, Knole, St. Paul's and the Tower of London. The Organising Committee has not overlooked the needs of the impoverished postgraduate who prefers cheap accommodation, for a block booking has been made of London University hostels. Full details are obtainable from the Conference Centre, 43 Charles Street, London, W.1. telephone 01-499-1101.

